# A systematic review and meta-analysis of surgeries performed for cerebral cavernous malformation-related epilepsy in pediatric patients

**DOI:** 10.3389/fped.2022.892456

**Published:** 2022-09-06

**Authors:** Xiangyu Gao, Kangyi Yue, Jidong Sun, Zheng Fang, Yuan Cao, Boyan Zhao, Haofuzi Zhang, Shuhui Dai, Lei Zhang, Peng Luo, Xiaofan Jiang

**Affiliations:** ^1^Department of Neurosurgery, Xijing Hospital, Fourth Military Medical University, Xi’an, China; ^2^Reproductive Medical Center, Tangdu Hospital, The Fourth Military Medical University, Xi’an, China

**Keywords:** cerebral cavernous malformations, epilepsy, pediatrics, neurosurgery, meta-analysis

## Abstract

**Background:**

The clinical benefit of surgery for the treatment of cerebral cavernous malformation (CCM)-related epilepsy in pediatric patients is still controversial. Although surgical treatment of CCM-related epilepsy in children is widely recognized, the clinical benefits of controlling the seizure rate must be balanced against the risk of leading to perioperative morbidity.

**Methods:**

We conducted a comprehensive search to identify relevant studies via Ovid Medline, Web of Science and PubMed (January 1995–June 2020). The following search terms were used: “hemangioma, cavernous, central nervous system,” “brain cavernous hemangioma,” “cerebral cavernous hemangioma,” “CCM,” “epilepsy,” and “seizures.” The seizure control rate and the risk of postoperative adverse outcomes along with their 95% confidence intervals (CIs) were calculated.

**Results:**

A total of 216 patients across 10 studies were included in meta-analysis. The results showed that the control rate of epilepsy was 88% (95% CI: 76–95%). Four percent (95% CI: 2–10%) of the patients experienced temporary symptomatic adverse effects following surgical resection, and 3% (95% CI: 0–26%) of the patients developed permanent symptomatic adverse effects in the long-term follow-up after surgical excision of the CCMs. None of the patients died as a result of the CCMs or surgical treatment.

**Conclusion:**

Surgery is an effective and safe treatment for CCM –related epilepsy in pediatric patients with a low risk of postoperative complications and death.

## Introduction

Cerebral cavernous malformations (CCMs) are cavernous vascular masses composed of monolayer endothelial cells in the brain parenchyma. CCMs account for 10–20% of all vascular lesions in the brain, with an incidence of 0.10–0.50% (1, 2). The incidence in children ranges from 0.30 to 0.53%. Children with CCMs may have different clinical manifestations from adults, such as higher bleeding rates and a greater incidence of lesion enlargement (3, 4). Twenty-five percent of children with CCMs may have epilepsy, hemiplegia, headache and other symptoms, even multiple acute bleeding, resulting in a high death and disability rate (5). The lesion site determines the patient’s clinical symptoms. When the lesion is located in the cerebral hemispheres, the patient often presents with epilepsy, which is the most common symptom of CCMs and seriously affects the children’s growth and life (6).

CCMs grow slowly, their incidence rate is low, and their natural history is not very clear; therefore, the ideal treatment method has not been determined. At present, the main treatment strategies for CCM-related epilepsy in children include conservative treatment, surgical treatment and radiotherapy. The clinical benefit of surgery is still controversial. Surgical treatment of CCM-related epilepsy in children is widely recognized, but the clinical benefits of controlling the seizure rate must be balanced against the risk of leading to perioperative morbidity. Therefore, we performed a systematic review and meta-analysis to investigate the clinical benefits of surgical treatment of CCM-related epilepsy in children.

## Methods

The present meta-analysis followed the Preferred Reporting Items for Systematic Reviews and Meta-Analyses (PRISMA) guidelines (7) and this study has been registered in PROSPERO (CRD42020206058).

### Search strategy

**FIGURE 1:**
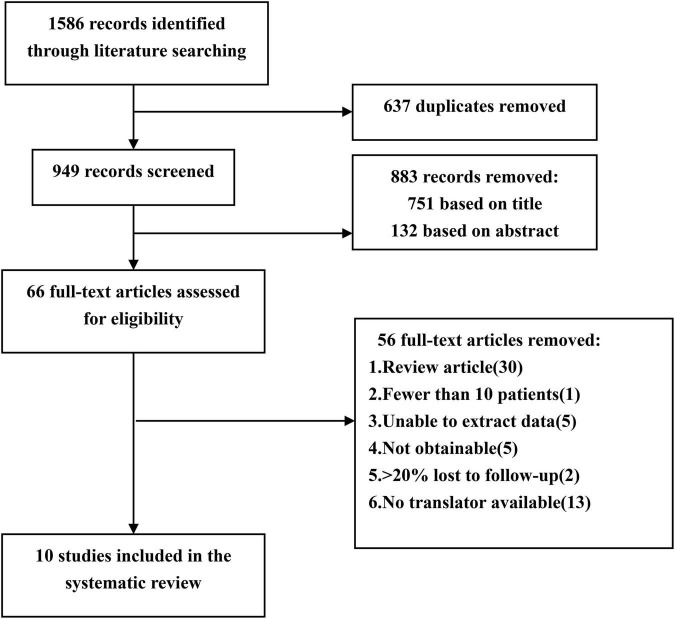
Flow chart of the data search followed by PRISMA guidelines. PRISMA, Preferred Reporting Items for Systematic Reviews and Meta-Analyses.

A comprehensive literature search was performed on Ovid Medline, Web of Science and PubMed (January 1995–June 2020). The search terms were used: “hemangioma, cavernous, central nervous system,” “brain cavernous hemangioma,” “cerebral cavernous hemangioma,” “CCM,” “epilepsy,” and “seizures.” We searched for original articles from cohort studies published in peer-reviewed journals. We included eligible studies published in English and Chinese, while studies published in other languages were excluded because we were unable to perform translations (Figure 1).

### Assessment of eligibility

Eligible studies were selected based on the Patient, Intervention, Comparison, Outcome, and Study design (PICOS) guidelines (8) by two independent reviewers: (1) Participants: pediatric patients with epilepsy symptoms before surgery should be confirmed by MRI or pathological examination; (2) interventions: neurosurgery; (3) comparison: not applicable; (4) outcome: seizure outcome estimated by Engel’s classification, temporary and permanent symptomatic adverse effects rate; (5) study designs: retrospective cohort study; the study must describe the duration of follow-up and the follow-up rate must be greater than 80%; the sample size of the study must be greater than 10. If the institution or author published multiple studies using the same cohort, only the report with the largest sample size was included for analysis. Letters, case reports, conference articles, meta-analyses and reviews were excluded.

### Risk of bias assessment

The quality of the included studies was assessed using the Newcastle-Ottawa Scale (NOS). The NOS score was used to assess three main factors: selection, comparability, and exposure. Studies with a score of ≥5 were defined as high-quality studies. Two reviewers independently assessed the quality of the study and resolved differences through discussion.

### Data extraction

A preliminary identification of 1,586 documents were carried out by this method. Two researchers (Xiangyu Gao and Peng Luo) independently extracted data and reviewed all articles. First, two researchers screened the titles and abstracts of the retrieved literature. They then evaluate the full text to determine eligibility. If the two researchers cannot reach an agreement, a senior researcher (Xiaofan Jiang) will be consulted. Finally, 10 out of 1,586 articles met the inclusion criteria (4, 9–17). The two researchers extracted the following data from the 10 studies: publication date, first author’s last name, total number of patients, number of female patients, mean age at surgery, mean duration of follow-up, mean duration of seizure, number of patients with drug-resistant epilepsy, type of epilepsy, number of patients with multiple CCMs, lesion location, postoperative seizure outcome, case fatality, and temporary and permanent symptomatic adverse effects (Table 1). The term “case fatality” is defined as the death of a patient due to CCMs or treatment. Temporary symptomatic adverse effects included new or worsened neurological deficits, transient brain edema after surgery, and a range of other complications, all of which could eventually completely recover. Permanent symptomatic adverse effects included memory decrease, persistent focal neurological deficits and so on.

**TABLE 1:** Basic patient characteristics of each included cohort.

References	Multi-center	Number of treated patients	Number of female patients (%)	Mean age at surgery (years)	Mean duration of seizure (years)	Mean duration of follow-up (years)	Drug-resistant epilepsy (%)	Type of epilepsy (%)	Number of patients with multiple CCMs (%)	Lesion location (%)					Engel class I (%)	Engel class II–IV (%)	Case fatality (%)	Symptomatic adverse effects (%)	
										T	F	P	Oc	Ot				Permanent	Temporary
Giulioni et al. (9)	N	11	5 (45.5)	12.5	1.6	5.6	11 (100.0)	36% GTCS, 36% SPS, 28% CPS	NA	NA	NA	NA	NA	NA	NA	NA	0.0	0.0	0.0
Consales et al. (4)	N	11	5 (45.5)	7.3	NA	4.0	NA	NA	3 (27.3)	36.4	36.4	22.7	4.5	0.0	NA	NA	0.0	0.0	0.0
Hugelshofer et al. (10)	Y	36	NA	9.7	NA	NA	12 (33.3)	NA	NA	NA	NA	NA	NA	NA	26 (72.2)	10 (27.8)	0.0	NA	NA
Gross et al. (11)	N	48	NA	NA	NA	NA	NA	NA	NA	NA	NA	NA	NA	NA	46 (95.8)	2 (4.2)	NA	NA	NA
Moraes Amato et al. (12)	N	16	5 (31.3)	7.6	NA	3.9	NA	NA	3 (18.8)	31.3	37.5	12.5	12.5	6.2	15 (93.8)	0 (0)	0.0	0.0	0.0
Noh et al. (13)	N	13	5 (38.5)	9.2	NA	NA	NA	77% GTCS, 23% SPS,	3 (23.1)	19.2	46.2	11.5	15.4	7.7	13 (100)	0 (0)	0.0	0.0	7.7
von der Brelie et al. (14)	N	22	8 (36.4)	13.9	2.3	10.7	8 (36.4)	NA	6 (27.3)	25.6	39.5	16.3	7.0	11.6	NA	NA	0.0	13.6	4.5
Sawarkar et al. (15)	N	17	5 (29.4)	13.1	2.3	4.9	NA	59% GTCS, 18% SPS, 23% CPS	NA	29.4	61.8	2.9	5.9	0.0	16 (94.1)	1 (5.9)	0.0	NA	NA
Lin et al. (16)	N	27	15 (55.6)	15.0	2.3	6.3	12 (44.4)	19% FAS, 48% FIAS, 33% FTBTCS	1 (3.7)	48.1	33.3	7.4	7.4	3.8	21 (77.8)	6 (22.2)	0.0	14.8	7.4
Aslan et al. (17)	N	15	9 (60.0)	12.3	NA	2.1	NA	47% GTCS, 33% SPS, 20% CPS	1 (6.7)	47.1	29.4	17.6	5.9	0.0	10 (66.7)	5 (33.3)	0.0	0.0	6.7

CCMs, cerebral cavernous malformations; T, temporal; F, frontal; P, parietal; Oc, occipital; O, others; N, no; Y, yes; NA, unknown; GTCS, generalized tonic-clonic seizures; SPS, simple partial seizures; CPS, complex partial seizures; FAS, focal aware seizure; FIAS, focal impaired awareness seizure; FTBTCS, focal to bilateral tonic-clonic seizures. We used median, if mean was not available.

### Statistical analysis

Seizure outcome data were estimated using the Engel’s classification. Engel class I represented no disabling seizure or aura only, and Engel class II–IV represented a seizure-free state. In order to standardize the evaluation of the study results, we performed a statistical analysis of the proportion of Engel class I patients and the proportion of patients with symptomatic adverse effects. The overall proportions were calculated using meta-analysis software (version 4.0.1, R). The statistical heterogeneity of the study results was assessed by the I^2^ statistic. If *I*^2^ > 50%, we used a random-effects model to analyze the hypothesis. Otherwise, we use a fixed-effects model. Publication bias was qualitatively assessed by funnel plot regression and quantitatively by Egger’s tests. When the *p*-value was < 0.1, Egger’s test considered publication bias to be statistically asymmetric. Sensitivity analyses were performed using Stata 14.2 to investigate the impact of a single study on the overall risk assessment by omitting one study per round.

## Results

### Systematic literature review

Ten studies were identified after screening, involving 216 pediatric patients (4, 9–17). All 10 studies were published between 1995 and 2020. One (10%) cohort was from a multicenter study, and the remaining 9 (90%) cohorts were from single centers. Three (30%) cohorts were from Asia, 5 (50%) cohorts from Europe, 1 (10%) cohort from North America and 1 (10%) cohort from South America. Seven studies (70%) described CCM lesion locations, and 7 (70%) studies described postoperative seizure outcomes with Engel’s classification. Nine (90%) studies reported postoperative case fatality, and 7 (70%) studies reported complications and symptomatic adverse effects.

### Results of meta-analysis

**FIGURE 2:**
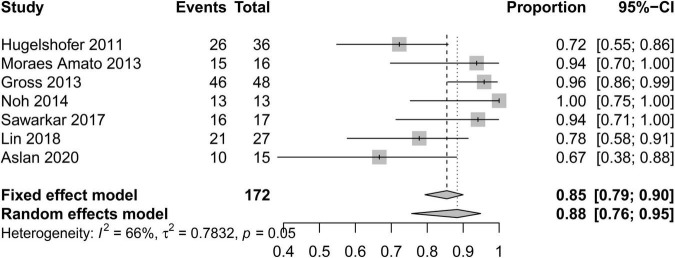
Forest plot of the seizure controlling following the surgical resection of CCMs in children.

**FIGURE 3:**
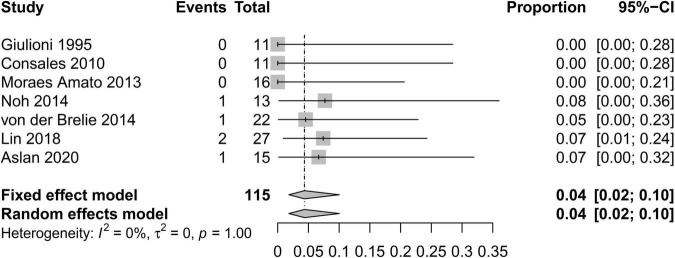
Forest plot of the percentage of temporary symptomatic adverse effects following the surgical resection of CCMs in children.

**FIGURE 4:**
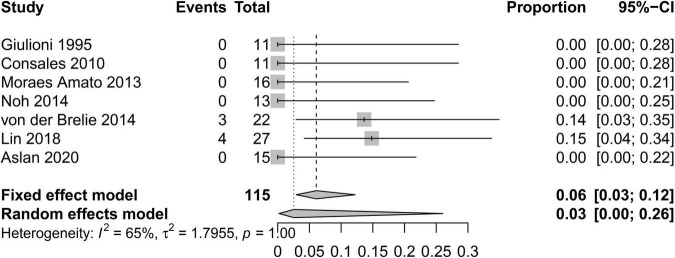
Forest plot of the percentage of permanent symptomatic adverse effects following the surgical resection of CCMs in children.

In order to standardize the evaluation of the study results, we performed a statistical analysis of the proportion of Engel class I patients and the proportion of patients with symptomatic adverse effects (Figures 2–4). As shown in Figure 2, the overall proportion of Engel’s class I CCMs was 88% (95% CI: 76–95%) in the 10 cohort studies, indicating that neurosurgery could significantly control seizures. Since *I*^2^ = 66%, we used a random-effect model to analyze the hypothesis. As shown in Figure 3, the fixed (*I*^2^ = 0%) pooled percentage of temporary symptomatic adverse effects following surgical resection was 4% (95% CI: 2–10%). In addition, 3% (95% CI: 0–26%) of the patients developed permanent symptomatic adverse effects during the long-term follow-up following the surgical resection of CCMs in children (Figure 4). Because I^2^ = 65%, we used a random-effect model to analyze the assumption. Case fatality was described in 9 (90%) studies, and the results showed that no one died from the CCMs or neurosurgery.

### Sensitivity analysis

**FIGURE 5:**
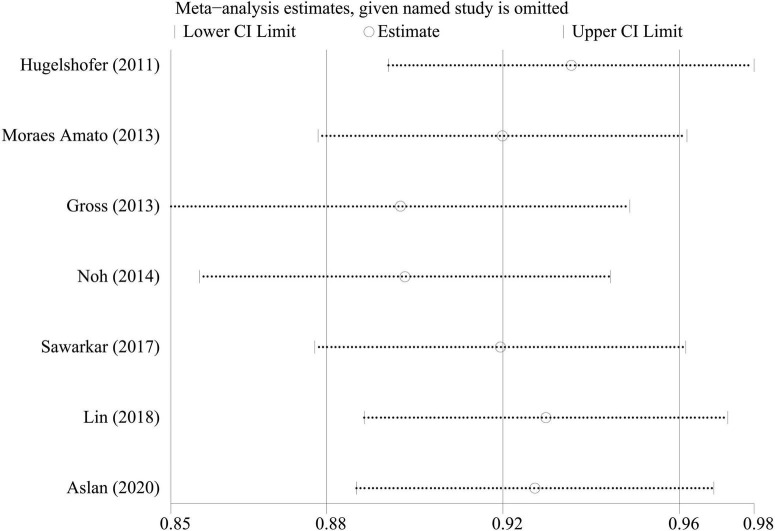
Sensitivity analysis of seizure controlling.

**FIGURE 6:**
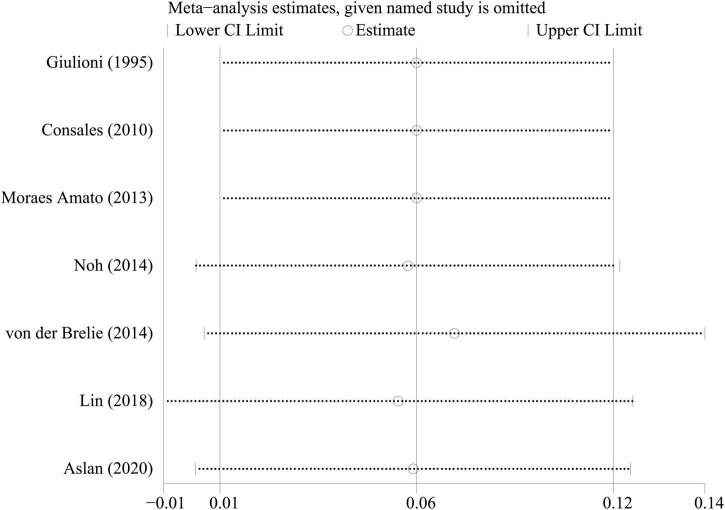
Sensitivity analysis of temporary symptomatic adverse effects rate.

**FIGURE 7:**
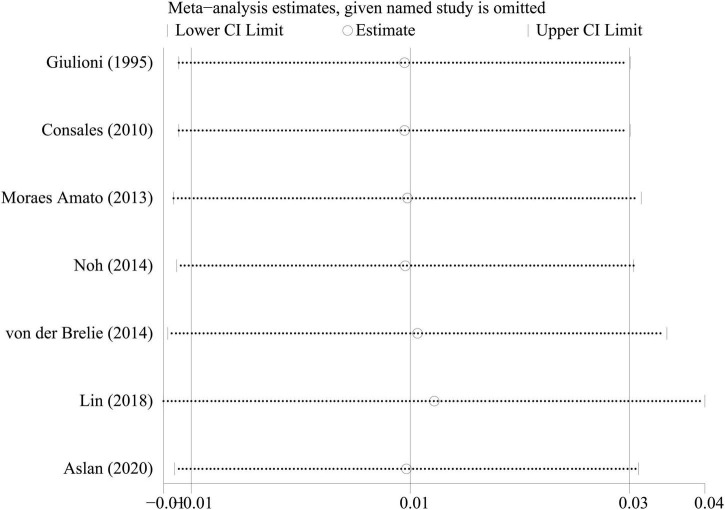
Sensitivity analysis of permanent symptomatic adverse effects rate.

To investigate the effect of a single study on the pooled rates, we omitted one study in each round. The comparison results did not change significantly, indicating that our results were statistically robust (Figures 5–7).

### Publication bias

**FIGURE 8:**
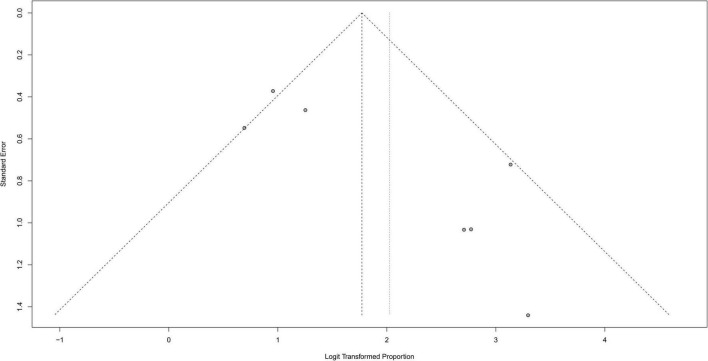
Funnel plot illustrating meta-analysis of seizure controlling.

**FIGURE 9:**
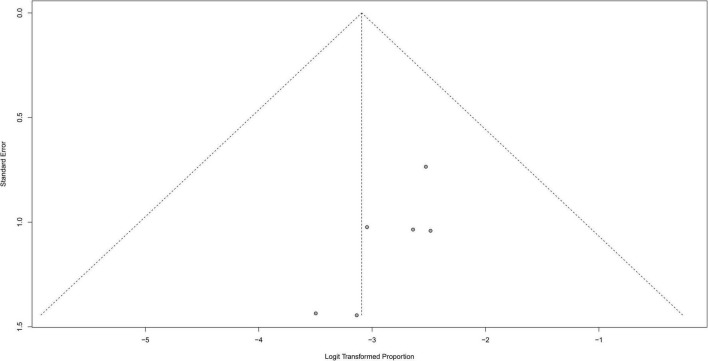
Funnel plot illustrating meta-analysis of temporary symptomatic adverse effects rate.

**FIGURE 10:**
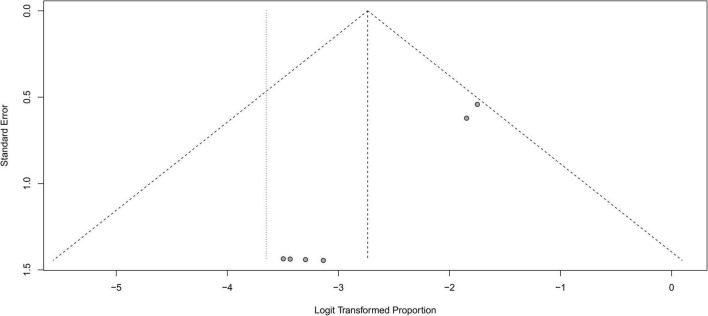
Funnel plot illustrating meta-analysis of permanent symptomatic adverse effects rate.

A funnel plot was used to evaluate the publication bias of the literature (Figures 8–10). The results of funnel plot analysis did not show any evidence of apparent asymmetry. The number of studies (*n* = 7) was too small, so Egger’s test could not be implemented.

## Discussion

CCMs are congenital vascular malformations composed of vascular clusters, which may be asymptomatic for a long time or produce clinical manifestations (9). CCMs account for 10–20% of all vascular lesions in the brain (1, 2). Approximately 25% of CCMs are diagnosed in children (18). The CCMs in patients under 18 years old differ from those of adults in origin and clinical characteristics. Asymptomatic lesions are more difficult to detect in children than in adults. The reasons are as follows: CCMs are acquired or growing lesions that are not easily found in childhood, but with increasing age, the lesion continues to grow and is thus more easily found in adults. Non-specific neurological disorders, such as chronic headache, are less common in children than in adults, and children are less likely to be tested for non-specific symptoms not associated with the malformation. The incidence of CCMs is higher in children aged 0–2 and 13–16 years (19, 20). In pediatric patients, the most common manifestation of CCMs in the CNS is partial or systemic epilepsy, some cases of which are refractory to medication. Mottolese et al. found that the type of epileptic symptoms in children was not related to the size of the lesion but was significantly related to the type of lesion. Lesions with severe calcification are more likely to produce epileptic manifestations, while lesions with large iron lutein rings are not associated with epileptic symptoms (18). In addition, imaging studies showed that temporal lesions were mainly responsible for epileptic seizures, and parsellar lesions could be accompanied by acute hemiplegia or epileptic events.

The incidence of epileptic seizures in pediatric patients is significantly higher than that in adults (21, 22). Due to the clinical use of magnetic resonance imaging (MRI) and because most previous studies treated both adult and child patients together, the natural history and treatment of CCMs has been well documented in adults but is still poorly understood in children. A large number of studies have recently been published focusing on pediatric patients with epilepsy induced by CCMs and reporting treatment modalities and efficacy. Approximately 15 years ago, CCM surgery was performed primarily in patients with chronic seizures who did not respond well to anticonvulsant therapy or to medication (intractable seizures), and surgical removal of CCMs was not strictly recommended for patients with well-controlled epilepsy via medication (23). Currently, the concept of epilepsy control in CCM patients, especially in children, has changed, and the decision to perform surgery depends on the balance of its benefits and risks. In the most recent pediatric series involving patients with CCMs, few children with chronic seizures were described because doctors often consider a clear treatment plan only when the clinical symptoms of epilepsy are present (24). Early surgical excision of CCMs in a superficial or non-critical location that causes seizures can prevent psychosocial disability in patients on long-term medication and can avoid the risk of growth of neurological defects (25, 26). Surgery can also improve the efficacy of anticonvulsant therapy in patients with drug-resistant epilepsy (9, 27, 28). Some scholars have recommended that the hemosiderin capsule around the CCMs be removed to avoid persistent epileptic seizures that may result from the stimulation of iron derivatives (9, 24, 29). However, some authors consider this dangerous because of the difficulty in distinguishing between hemosiderin-stained brain tissue and the surrounding atrophic nerve tissue (30, 31).

The length of preoperative seizure history has an important influence on the prognosis of CCMs in pediatric patients. With a longer history of seizures before surgery, the likelihood of persistent seizures after surgery increases (31). Pediatric patients have a longer life expectancy. Children with symptomatic and rapidly growing CCMs should be aggressively treated as soon as they are diagnosed. Early surgery in pediatric patients can help prevent the adverse effects of epilepsy on intellectual and cognitive development. Moreover, when considering the harmful side effects and high lifetime costs of antiepileptic drugs, seizure control should be a relative indication for surgery. The decision to perform surgery on pediatric patients with CCMs must take into account both the clinical benefits and the potential neurological deficits associated with the surgery.

To determine the clinical benefits of surgery for CCM-related epilepsy in children, we conducted a systematic review and meta-analysis of available data from the published literature. The results of our meta-analysis showed that postoperative epilepsy was effectively controlled in 88% (95% CI: 76–95%) of patients. Four percent (95% CI: 2–10%) of the patients experienced temporary symptomatic adverse effects following surgical resection, and 3% (95% CI: 0–26%) of the patients developed permanent symptomatic adverse effects during the long-term follow-up following the surgical resection of CCMs. None of the patients died from the CCMs or surgical treatment. Our data indicated that neurosurgery is a significantly effective treatment for CCM -related epilepsy in pediatric patients with a low risk of postoperative complications and death. Some researchers found long-term conservative treatment could increase the incidence of mental illness and neurological defects because of the long life expectancy of pediatric patients (25, 26). Compared with conservative treatment, surgical treatment could not only cure epilepsy, especially drug-resistant epilepsy, but also reduce the incidence of mental illness and neurological defects. Additionally, in the past few decades, with the application of advanced technology such as neuronavigation systems, intraoperative neuromonitoring, MRI, functional MRI and brain mapping, surgical intervention has been able to yield better results (32–34).

Six of the included studies documented pediatric patients with multiple CCMs. Multiple CCMs usually have a genetic background and are common in patients with familial CCM. Familial CCMs, which tend to show clinical symptoms in younger patients, are more suitable for early surgical resection (35).

At present, radiotherapy is also often used to treat CCMs. Radiotherapy can shrink and block the sinus and reduce the volume of CCMs, but there are few reports on radiotherapy as a treatment option for pediatric patients. Di Rocco et al. determined that the effect of radiotherapy was not very certain and that the side effects on children were relatively large; therefore, they could not generally recommend the treatment (36). Scott et al. also believed that even stereotactic radiotherapy with small surgical fields and gamma-knife radiotherapy was not appropriate for children (37). Additionally, rare studies demonstrated the usefulness of endovascular treatment in CCMs. Yoshimura et al. reported a rare case of giant hypervascular CCMs in pediatric patient that was treated with preoperative endovascular embolization followed by successful total resection (38). The ARUBA trial (A Randomized Trial of Unruptured Brain Arteriovenous Malformations) confirmed that the risk of permanent neurological dysfunction and treatment-related death was significantly lower in the surgical group than in the other treatment options (39). This trail also demonstrated the value of preoperative embolization as an adjunct to reduce programmed bleeding by managing the associated aneurysm and reducing blood flow into the nidus. CCMs is likely to present with massive intraoperative hemorrhage. Therefore, preoperative embolization could be very useful for safe maximal resection in pediatric patients. More research on endovascular treatment is urgently needed.

We used NOS to evaluate the quality of the 10 studies, and each study was of moderate quality with an average score of 5. There were four limitations in our systematic review and meta-analysis. First, all the included cohorts were retrospective cohorts. Second, the type of neurosurgery and the surgeon’s experience were not consistent across all included studies. Third, the Engel’s classification was the only indicator we used to evaluate the control of epilepsy. Finally, most studies did not provide information on the duration of epilepsy before surgery and on medication regimens. Though these issues were not addressed in our study, but will certainly contribute to excellent questions in future investigations.

## Conclusion

Taken together, our study indicates that surgery is an effective and safe treatment for CCM -related epilepsy in pediatric patients with a low risk of postoperative complications and death.

## Data availability statement

The original contributions presented in this study are included in the article/supplementary material, further inquiries can be directed to the corresponding author/s.

## Author contributions

XG, KY, and JS contributed to the conception and design of the study. PL and XJ organized the database. ZF and YC performed the statistical analysis. XG wrote the first draft of the manuscript. BZ, HZ, SD, LZ, and PL wrote sections of the manuscript. All authors contributed to the manuscript revision and read and approved the submitted version.
